# Effective In Vitro Control of Two Phytopathogens of Agricultural Interest Using Cell-Free Extracts of *Pseudomonas fluorescens* and Chitosan

**DOI:** 10.3390/molecules26216359

**Published:** 2021-10-21

**Authors:** Ariadna Berenice Trejo-Raya, Víctor Manuel Rodríguez-Romero, Silvia Bautista-Baños, Francisco Roberto Quiroz-Figueroa, Ramón Villanueva-Arce, Enrique Durán-Páramo

**Affiliations:** 1 Laboratory of Food Biotechnology, Unidad Profesional Interdisciplinaria de Biotecnología (UPIBI), Instituto Politécnico Nacional (IPN), Av. Acueducto s/n, Barrio La Laguna Ticomán, Ciudad de México CP 07340, Mexico; berenice.trejo.r@gmai.com (A.B.T.-R.); rarce@ipn.mx (R.V.-A.); 2 Centro de Desarrollo de Productos Bióticos (CeProBi), Department of Postharvest Technology, IPN, Km 8.5 Carretera Yautepec-Jojutla, San Isidro, Yautepec CP 62731, Morelos, Mexico; sbautist@ipn.mx; 3 Laboratory of Molecular Plant Breeding, Centro Interdisciplinario de Investigación para el Desarrollo Integral Regional Unidad Sinaloa (CIIDIR-IPN Unidad Sinaloa), Juan de Dios Batiz Paredes No. 250, San Joaquin, Guasave CP 81101, Sinaloa, Mexico; fquiroz@ipn.mx

**Keywords:** *Alternaria altenata*, *Fusarium solani*, siderophores, antagonist, biofungicide

## Abstract

A biofungicide is a natural product that can be derived from various sources such as, among others, microorganisms, higher plants, animal products, phytochemicals, semiochemicals, and antagonist microorganisms. One of the most important approaches for the production of biofungicides is the combination of biocontrol agents. This study showed the inhibition growth of *Alternaria alternata* and *Fusarium solani* treated with cell-free extracts of *P. fluorescens*. Using thin-layer chromatography and plate assays it was also demonstrated that the cell-free extracts of *P. fluorescens* contained siderophores and derivates of 4-diacetylphloroglucinol and phenazine. Moreover, the combination of cell-free extracts of *P. fluorescens* and chitosan [50–1.5% (*v*/*v*)] had a synergistic effect since they notably inhibited the mycelial growth of *A. altenata* and *F. solani*. Various morphological alterations to the mycelia and conidia of the treated fungi as a result of this combination were also observed. The present study could be a starting point to control other fungal phytopathogens using different cell-free extracts and chitosan as biocontrol agents.

## 1. Introduction

Phytopathogens are microorganisms that cause plant diseases and metabolic disorders [1] and are the main cause of crop diseases throughout the world. More than 19,000 varieties of phytopathogens have contributed to complete crop devastations and have caused losses worth millions of dollars [2,3]. It has been noted that approximately 8000 fungal species have caused nearly 100,000 diseases in plants [4]. The most harmful and significant phytopathogen species are found primarily in the phyla Ascomycota and Basidiomycota [5]. Until now, the fungal genera that play an important role in agriculture are *Botrytis, Fusarium, Alternaria, Colletotrichum, Magnaporthe*, and *Cladosporium* [6,7]. Although the primary approach to controlling them has been the use of synthetic fungicides, not all fungal diseases can be adequately controlled in this way.

In addition, most synthetic fungicides are known to be toxic to humans and have adverse environmental effects on soil, animals, and plants [8]. It is widely reported that their continuous use has disrupted natural biological systems and has given rise to a lack of response in fungal pathogens to fungicides. To tackle this problem, the industry and academia have developed and studied other alternatives, such as the development of biological-based products [9,10,11,12].

A biofungicide is a biological control product that can include the use of living organisms and their derivatives (metabolites, extracts, etc.) without presenting adverse effects in humans [13]. One of the most important approaches for biofungicides is the biocontrol plant growth-promoting rhizobacteria (PGPR). These are facultative intracellular endophytes that exert beneficial effects in plants through direct and indirect mechanisms capable of suppressing or preventing pathogens by antibiosis [14,15]. The best characterized biocontrol PGPR belongs to the bacteria genus *Pseudomonas* [16].

The specie *Pseudomonas fluorescens* is a PGPR bacteria that has demonstrated efficient biocontrol in vitro against fungal phytopathogens through the action mechanism of antibiosis [17,18], including secondary metabolites, the foremost of which are 2,4-diacetylphloroglucinol (DAPG); siderophores; phenazines (Ph); and hydrogen cyanide [19]. However, its performance in field conditions is weak and variable because of the loss of viability when stored for several weeks [20]. One quick way to address this issue is by using cell-free extracts, which have extracellular metabolites that can be recovered by the culture medium, and which can exert antifungal activity by putting aside the biomass [21].

On the other hand, when different methods are applied in combination, e.g., mixtures of living microorganisms, cell-free extracts, volatile extracts, or biological derivatives, as well as non-synthetic antifungal substances, such as chitosan, among others [22,23,24], an array of action mechanisms can be triggered with regard to the phytopathogens.

Chitosan is a biopolymer that can retard or control the growth of several microorganisms that cause a severe disease at pre- and post-harvest stages [25]. The fungicidal capacity of this compound can be correlated with its the concentration; for example, a nutritious medium supplemented with high concentrations of chitosan can inhibit the growth of the mycelia of different phytopathogens [26,27]. Furthermore, this antimicrobial capacity of chitosan could be enhanced with the addition of bacterial extracts, which have different mechanisms of action through metabolites with regard to inhibitory activity.

The main objectives of this study were to evaluate the effect of cell-free extracts of *P. fluorescens* and chitosan alone and combined on the in vitro development of *Alternaria alternata* and *Fusarium solani*, to identify the possible secondary metabolites present in the cell-free extracts of *P. fluorescens*, and to observe the effect of the mixture of *P. fluorescens* and chitosan in the fungal morphology of both phytopathogens.

## 2. Results

### 2.1. Culture Kinetics of P. fluorescens and the Cell-Free Extracts of Antifungal Activity of P. fluorescens

**Figure 1:**
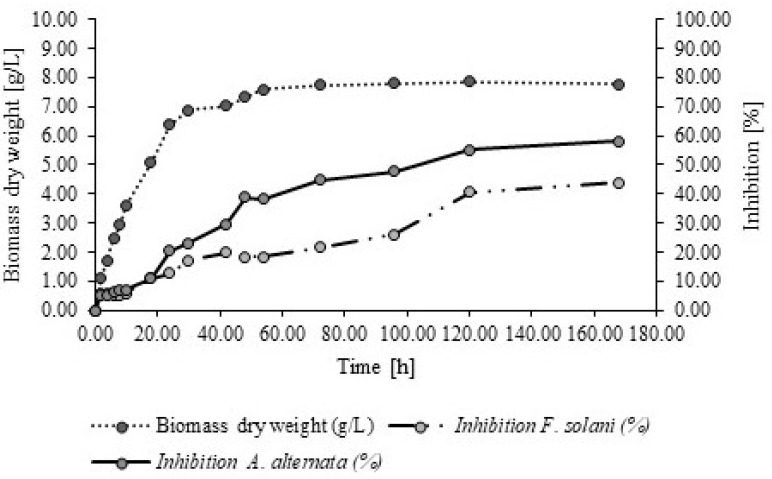
Growth kinetics of *P. fluorescens* and the antifungal effect of cell-free extracts of *P. fluorescens* on *A. alternata* and *F. solani* incubated for 168 h.

The bacterium P. *fluorescens* reached the exponential phase at 42 h, where the specific growth rate (μmax) was μ = 0.1989 h^−1^, while the stationary phase was at 72 h (Figure 1). Also, overall, the mycelial growth of both tested fungi was reduced as the biomass of *P. fluorescens* increased (Table 1). Finally, the highest antifungal activity of the cell-free extracts of P. *fluorescens* to inhibit *A. alternata* and *F. solani* coincided with the stationary phase period of *P. fluorescens* at 72–168 h (Figure 1). The antifungal activity of the studied fungi was accordingly linked with the production of the biomass of *P. fluorescens,* inferring that there are secondary metabolites produced during the stationary phase which are involved in the antifungal activity during growth.

As shown in Table 1, the fungicide Captan^®^ and the cell-free extracts of *P. fluorescens* show a more significant reduction of mycelial growth on *A. alternata* from 42 h onwards (*p* < 0.05), while for *F. solani* this outcome was after 54 h (*p* < 0.05). In this context, it was essential to identify the possible secondary metabolites present in the cell-free extracts of *P. fluorescens*.

### 2.2. Identification of Antifungal Metabolites in Cell-Free Extracts of P. fluorescens

As shown in Figure 1 and Table 1, the cell-free extract of *P. fluorescens* showed the highest antifungal activity in both fungi after 168 h of incubation; therefore this 168 h of incubation time was used as a reference for identifying the secondary metabolites of *P. fluorescens.*

**Figure 2:**
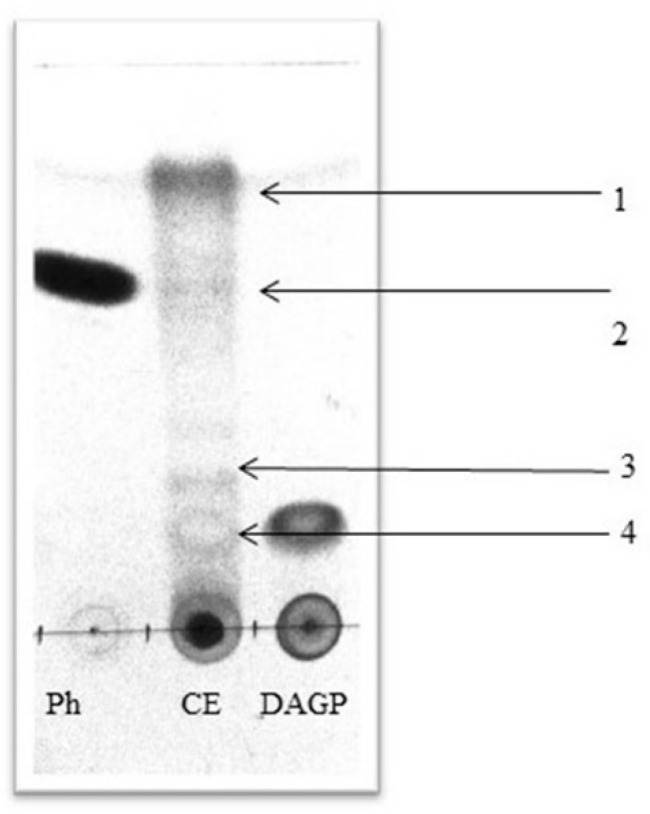
Readings of the TLC. Ph = Standard phenazine, CE = cell-free extracts of *P. fluorescens* and DAGP = standard of for 2,4-diacetylphloroglucinol. Readings are on UV 245 nm. Numbers 1, 2, 3, 4 indicate the possible secondary metabolites with antifungal effects.

The chromatography results showed at least four compounds which were compared with the standards of phenazine (Ph) and 2,4-diacetylphloroglucinol (DAGP) (Figure 2).

The readings of TLC (Table 2) could indicate that the cell-free extract of *P. fluorescens* contains at least four antifungal compounds that could confer the antifungal activity derived from Ph and DAGP, secondary metabolites involved in the biocontrol process.

**Figure 3:**
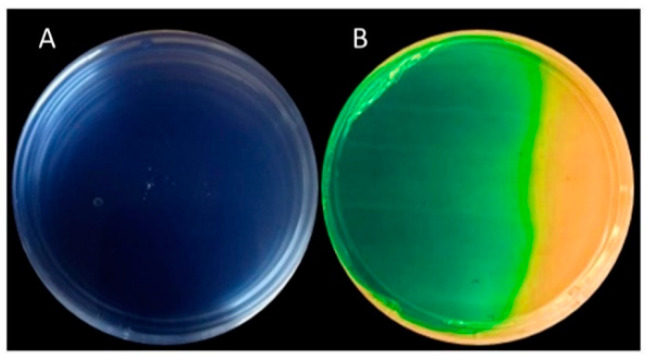
Petri plate with sterile distilled water as a control (**A**) and cell-free extracts of *P. fluorescens* (**B**). The scale of color is as follows for siderophore production: blue = none, green (blue + pale yellow) = limited, yellow = very strong.

Other secondary metabolites were siderophores, which are reported as one of the main biocontrol compounds for *P. fluorescens.* The most important are pyoverdin and pyochelin. According to CAS plate assay [28], the production of siderophores from the cell-free extracts of *P. fluorescens* led to a change from blue to an intense yellow color (Figure 3).

### 2.3. Combination of Chitosan and Cell-Free Extract of P. fluorescens and Its Antifungal Activity

The results showed that with exception of the distilled water, all treatments exhibited antifungal properties (Table 3); however, for both tested fungi, the highest antifungal activity was for the cell-free extracts of *P. fluorescens* and the combination of cell-free extracts with chitosan [50–1.5% (*v*/*v*)].

### 2.4. Effect of the Combination of Chitosan and Cell-Free Extract of P. fluorescens on the Morphology of Mycelia and Conidia of A. alternata and F. solani

**Figure 4:**
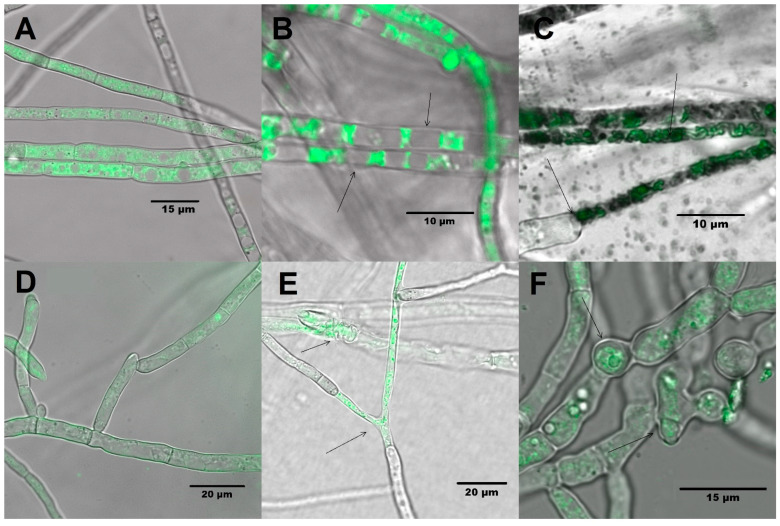
Mycelia of *A. alternata* without treatment (**A**) and treated (**B**,**C**) with a mixture of cell-free extracts of *P. fluorescens* and chitosan [50–1.5% (*v*/*v*)]. Mycelia of *F. solani* without treatment (**D**) and treated (**E**,**F**) with the mixture of cell-free extracts of *P. fluorescens* with chitosan [50–1.5% (*v*/*v*)].

Figure 4A,D show that the non-treated hyphae of *A. alternata* and *F. solani* had a continuous shape; in contrast, it was observed in the treated fungi that there were hyphae containing a great vacuolization and formation of intracellular aggregates with swelling and shortening of the septa (Figure 4B,C,E,F).

**Figure 5:**
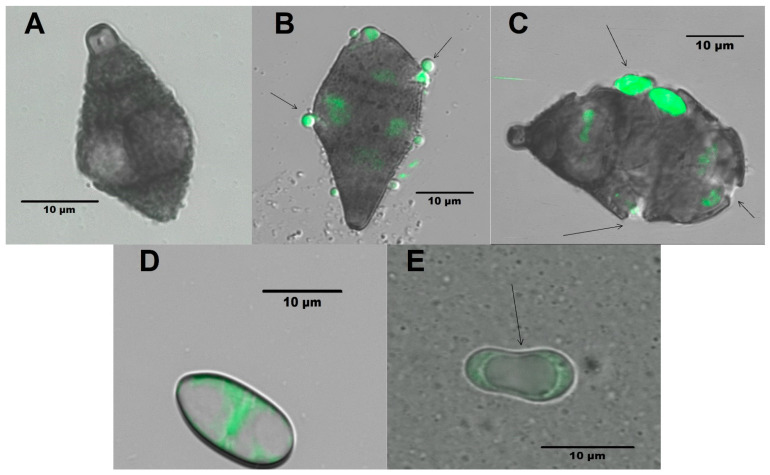
Conidia of *A. alternata* without treatment (**A**) and treated (**B**,**C**) with a mixture of cell-free extracts of *P. fluorescens* and chitosan [50–1.5% (*v*/*v*)]. Conidia of *F. solani* without treatment (**D**) and treated (**E**) with a mixture of cell-free extracts of *P. fluorescens* and chitosan [50–1.5% (*v*/*v*)].

With respect to the conidia of both treated fungi, as can be seen in Figure 5A,D, they are well-shaped and formed conidia, with the cell wall and membrane smooth and distinctive. In Figure 5B,C in the case of *A. alternata,* distorted conidia were observed. In Figure 5E, in the case of *F. solani,* it was noticed that there were intracellular material and cell wall external modifications.

## 3. Discussion

Many studies have characterized the antimicrobial properties of *P. fluorescens* and its potential as a biofungicide in field conditions. The main inconvenience is the use of biomass that is inconsistent with field conditions. However, an alternative could be the use of the cell-free extracts of *P. fluorescens.*

The effects of cell-free extracts of different bacteria have not been consistent; it depends on the action mechanism that presents the bacteria, and it is assumed that the action mechanism is related to the inclusion of secondary metabolites. Moreover, it is essential to determine the age of the culture when the cell-free extracts are obtained [29]. Hence, it is crucial to characterize the kinetics of the production of the cell-free extracts of *P. fluorescens* to determine when they can be used and when they have antifungal properties. The specific growth rate of P. fluorescens was 0.29 h^−1^ (these results are in line with previously reported results which ranged from 0.2173 to 0.2874 h^−1^ [30,31]).

The findings in this study confirm the antifungal properties of the cell-free extracts of *P. fluorescens*, with regard to the treated fungi, an effect that has been supported by previous studies [20]. The antifungal properties of the cell-free extracts of *P. fluorescens* are related to secondary metabolites [32], as shown in the growth kinetics, and in the production of secondary metabolites, that is often correlated with biocontrol activity [33]. Other results have demonstrated that aged (more than 3 days) bacterial cultures from biocontrol present good antifungal activity [34]. The cell-free extracts of *P. fluorescens* that showed that the higher antifungal activity on *A. alternata* and *F. solani* was related to the stationary phase, which in turn is related to the ceasing of the bacterial growth, being the most metabolically active period and when the production of secondary metabolites takes place [35].

It was found that the cell-free extract of *P. fluorescens* presented derivate compounds such as DAGP and Ph. The identification of these compounds coincided with previous findings regarding extracts of the same genus and species [36,37,38]. DAGP and Ph are broad-spectrum antibiotics consisting of phenolic molecules with antifungal, antibacterial, anthelmintic, and phytotoxic characteristics [39]. Currently, they are one of the main objects of study in biological control since they play a significant role in competition in the rhizosphere environment [40]. However, in field conditions, the presence of DAGP and Ph in the rhizosphere is limited by abiotic and biotic factors associated with the crop’s type, age, time of the year, etc. [41]. This situation results in weak and variable antifungal activity on the part of *P. fluorescens*. This limitation would be addressed using cell-free extracts because DAGP, Ph, and other secondary metabolites are already presented, and their production does not depend on biotic or abiotic factors.

The results obtained from TLC are supported by previous findings [42]. Different isolates of *P. fluorescens* were obtained from the rhizosphere of rice, showing notable antifungal activity against *Magnaporthe grisea, Dreschelaria oryzae, Rhizoctonia solani,* and *Sarocladium oryzae*. In that study, the metabolites produced by *P. fluorescens* showed four antifungal compounds corresponding to Rf 0.22, 0.35, 0.42, and 0.51, which are very similar to those found in this research.

The findings in the CAS plate assay agree with those expected with regard to strains of *P. fluorescens.* The production of siderophores by different strains of *P. fluorescens* is well documented [43,44,45]; the identified siderophores are pyoverdine and pyochelin [22,46,47]. The results of the present study showed the presence of siderophores in the cell-free extract of *P. fluorescens*. Therefore, their presence could be what is conferring the antifungal properties on the two studied fungi. However, more quantitative tests are needed to characterize their presence in the cell-free extract.

To increase the antifungal properties of the cell-free extracts of *P. fluorescens*, we propose the use of combination of bacterial extracts with chitosan, since the combination of more than one biocontrol agent involves more than one action mechanism exploiting the potential synergistic effects between them [48]. Chitosan is a suitable antimicrobial agent to combine with other agents. This compound is an agent that has been extensively studied and one which presents antimicrobial activity. So far, this polymer has been known to be very effective in inhibiting spore germination, germ tube elongation, and radial growth, among other antifungal properties [49]. The literature reports that the antifungal mechanism of chitosan is related to cell wall morphogenesis, with chitosan molecules interfering directly with the fungal growth [50]. Currently, chitosan is used as a vehicle to integrate natural products such as plant extracts, essential oils, acids, microorganisms, and cell-free extracts, among others [51]. The combination of chitosan with other agents enhances the synergistic effect [49,51].

Previous studies that showed the effective use of cell-free extracts of *P. fluorescens* with chitosan [50–1.5% (*v*/*v*)], agreed with this study’s findings. The synergistic effect could be due to the combination of two action mechanisms. The positive charge of chitosan interacts with the negatively charged phospholipid components of the fungi membrane, increasing the membrane’s permeability, and causing the leakage of cellular content [52]. This phenomenon could allow the entry of the secondary metabolites of the cell-free extracts of *P. fluorescens*, such as pyoverdine, pyochelin, and derivate compounds of DAGP and Ph, which present different action mechanisms. Together with cell-free extracts of *P. fluorescens* and chitosan, they act together and attack at different levels through different action mechanisms.

It is important to characterize how the combination of chitosan and cell-free extracts of *P. fluorescens* [50–1.5% (*v*/*v*)] affected the conidia and hyphae of *A. alternata* and *F. solani.* To the best of our knowledge, we provide the first evidence of the effects of both cell-free extracts of *P. fluorescens* and chitosan compound on fungi cellular morphology, although the effect of each separate component has been reported. The application of chitosan (1.0%) caused a slimming in the hyphae and swelling in *A. alternata* and *B. cinerea* [53]. In that study, it was also observed that the application of chitosan led to the formation of long vacuoles and empty spaces in the cytoplasm [54]. On the other hand, it has been observed that Ph obtained in the cell-free extracts of *P. chlororaphis* caused evident disorganization in the intracellular content and an abnormal swelling with regard to *Rhizoctonia solani* [55]. Hyphal shortening, loss of membrane integrity, and lysis of *M. phaseolina* and *S. sclerotiorum* caused by metabolites such as siderophores and antibiotics produced by a fluorescent *Pseudomonas* has been reported [56].

The phenomena mentioned, such as vacuolization, results from the fungus’s adaptation to toxic substances and to external agents. The organelles sequester and eliminate these substances in order to maintain the homeostasis of the cytoplasm [57]. The development of enlarged hyphal segments occurs when the fungi develop in an environment that restricts their growth. The formation of these fractions or “cells” could be morphologically equivalent to the sporophores in which the development of conidia is not completed due to metabolism disorders when antifungal agents are applied [58].

The slimming of the hyphae and the loss of cytoplasmic content are caused by the electrostatic interaction of the amino groups of chitosan with the negative charges of the membrane, increasing permeability and leading to the leakage of intracellular content [59]. This phenomenon increases the susceptibility of the fungus to external agents, and it could allow the entry of phenazines and pyocyanin (which are secondary metabolites commonly produced by *P. fluorescens).* These secondary metabolites diffuse through the membrane, causing reactive oxygen species production that subsequently induces apoptosis and death [60].

Our results could become a starting point for the use of different cell-free extracts from bacteria identified as biofungicides to control relevant fungi phytopathogens. Also, the combination of chitosan with cell-free extracts could be used to enhance the antifungal effects of both compounds. These findings can lead to the development a new biofungicide, which could compete with the current use of synthetic fungicides.

## 4. Materials and Methods

### 4.1. Biological Material

*Pseudomonas fluorescens* was isolated from strawberry stolons and identified culturally and molecularly at the Postgraduate Laboratory of Food Biotechnology at the National Polytechnic Institute in Mexico City. The culture was maintained in Agar King’s B. (agar K.B.). The fungal phytopathogen *A. alternata* was isolated from tomato fruit and *F. solani* was obtained from chili fruits with the typical symptoms of the disease. Both were identified and molecularly characterized [61] at the Postharvest Physiology Laboratory of the Center for the Development of Biotic Products at the National Polytechnic Institute. The fungi were cultivated in PDA (Bioxon, CDMX, Mexico) for 4–7 days and incubated at room temperature (25 ± 2 °C) for subsequent tests.

### 4.2. Growth Kinetics of P. fluorescens

*Pseudomonas fluorescens* was cultivated in King’s B medium (25 ± 2 °C, 120 rpm) and incubated for 7 days at 25 °C. Samples of 50 mL were taken at 2, 4, 6, 8, 10, 18, 24, 30, 42, 48, 54, 72, 96, 120 and 168 h, to determine the biomass parameters (dry weight and optical density), and antifungal activity (mycelial growth mm and inhibition %).

The growth of *P. fluorescens* was evaluated by dry weight and optical density. The cell growth was estimated turbidimetrically at 600 nm (UV-Vis Thermo Scientific™ GENESYS 10UV, Waltham, Massachusetss, EUA) and gravimetrically through a 10 mL sample centrifuged at 8000 rpm for 10 min. The supernatant was discarded, and the pellet was recovered in a foil pan (previously dried at 85 °C for 24 h and weighed). The dry weight was obtained through weight differences.

The specific growth rate (μmax) in the exponential phase was calculated [62] using the following Equation (1):

(1) $$\mu \max=ln\frac{BDW2-BDW1}{t2-t1}$$

where, μmax =maximum specific growth rate, BDW = biomass dry weight and *t* = biomass at different time points (*t*1 and *t*2, respectively).

### 4.3. Obtention of Cell-Free Extracts of P. fluorescens and Evaluation of its Antifungal Activity

The *P. fluorescens* extract was obtained after each of 30 mL sample was centrifuged for 10,000 rpm for 15 min at 4 °C. The pellet was discarded, and the supernatant was filtered through sterile membranes (Cole Palmer, Vernon, IL, USA) of 0.22 µm to collect the residual biomass. For the inhibition of mycelial growth assays, the experimental unit was a Petri dish (55 × 15 mm of diameter) with PDA. For this, a 0.25 mL sample was deposited and distributed uniformly on the surface medium and allowed to dry for 5 to 10 min. Then one disk (5 mm in diameter) of PDA with fungal mycelia was placed in the center of the box and incubated at room temperature. Distilled water as a negative control and Captan^®^ (0.25% *w*/*v*) as a positive control were also included. The incubation time limit was determined when the mycelial growth of the negative control reached the edge of the box. For each experimental unit, the diameter of the colony was measured and averaged in two directions. The percentage of mycelial inhibition [63] was calculated using Equation (2):

Inhibition (%) = [(DC − DT)/DC) × 100](2)

where DC is the diameter of the control culture, and DT is the diameter of the treatment culture. Six repetitions of this assay were considered.

### 4.4. Identification of Antifungal Metabolites in Cell-Free Extracts of P. fluorescens

The cell-free extract of *P. fluorescens* that showed the highest antifungal activity was identified with the use of TLC. The cell-free extract of *P. fluorescens* was mixed with ethyl acetate in a 1:1 ratio and stirred for 2 h at 120 rpm. After separating the aqueous layer, the organic fraction was again separated and evaporated under vacuum and dried at 60 °C for 24 h. The dried cell-free extract of *P. fluorescens* was resuspended in ethyl acetate at a concentration of 1 mg of extract/mL of ethyl acetate. Samples of 50 μL of phloroglucinol and phenazine standards (Sigma-Aldrich, St Louis, MO, USA) were subjected to thin-layer chromatography (TLC; silica gel. 60 F254, 20 × 20, 0.5 mm, Merck and Co, Inc, Branchburg, NJ, USA) with a solvent system hexane:acetone 3:2. The plate was read at 254 nm U.V. The siderophore was detected using the plate assay method [64]. The ternary complex chrome azurol S (CAS)/Fe^3+^/hexadecyltrimethylammonium (HDTMA) bromide complex served as an indicator. To prepare 1 L of the blue agar, 60.5 mg of CAS was dissolved in 50 mL of water mixed with Fe III (1 mM FeCl_3_: 6H_2_O in 50 mL of 10 mM HCl; 10 mL). The indicator was mixed with 900 mL of bacteriological agar (pH 7). The plates were inoculated with 1 mL of cell-free extract of *P. fluorescens*. Distilled water was included as a negative control. The plates were incubated for 10 days. The production of siderophores was classified as follows: none, small (light yellow), strong (moderate yellow) very strong (yellow). There were six repetitions of this assay.

### 4.5. Preparation of Chitosan and Evaluation of its Antifungal Activity

The chitosan solution (Sigma Aldrich, Sant Louis MO, USA) was prepared at 1.5% (*w*/*v*) and obtained by dissolving 1.5 g of chitosan of low molecular weight (50–190 kDa, 85% deacetylation) in 100 mL of distilled water to which glacial acetic acid (Fermont, Mexico City, Mexico) was slowly added at 1% (*v*/*v*). The solution was stirred and warmed to 40 °C for 24 h; the pH was adjusted to 5.6 with 1 N of sodium hydroxide and sterilized for 15 min at 15 psi. The antifungal activity of chitosan on mycelial growth was determined using previously described methodology. There were six repetitions of this assay.

### 4.6. Preparation of the Combination of Chitosan and Cell-Free Extract of P. fluorescens and the Evaluation of the Antifungal Activity

The preparation of chitosan and extracts of *P. fluorescens* (1.5–50% [*v*/*v*]) was done according to Rodriguez-Romero [20]. Both compounds were mixed together at a concentration of 50–50% *v*/*v*. The antifungal activity of the combined compounds on mycelial growth and conidial germination was determined using the same methodology mentioned above (Section 4.3). There were six repetitions of this assay.

### 4.7. Effect of the Combination of Chitosan and Cell-Free Extract of P. fluorescens on the Morphology of Mycelia and Conidia

To evaluate the morphological changes, a sample of mycelium was taken from the hyphal tips of 3 Petri plates of the tested fungi, and the methodology described by Hickey et al. [65] was followed with the samples being placed on separate slides. After, 20 µL of a membrane staining solution in dimethylsulfoxide (FM4-64^®^ Lipophilic Styryl Dyes-ThermoScientific, Waltham. IL, USA) was added at a concentration of 5 µM for 1 min, placed on each slide, and observed using a Leica TCS SP5X confocal fluorescence microscope (KEYENCE, Itasca, IL, USA) with a 40X immersion objective with excitation/emission 515/630–650 nm. For the assay on the conidia of *A. alternata* and *F. solani*, 3 microtubes were inoculated separately. Containing a double concentration of potato dextrose broth (PDB) together with a combination of cell-free extracts of *P. fluorescens* and chitosan after 24 h incubation at room temperature, the samples were observed following a similar methodology to that used for mycelia morphology.

### 4.8. Statistical Analysis

For in vitro assays, a completely randomized design was used. A normality test was performed. ANOVA was used to analyze the data, and the treatments were compared using the Tukey test (* = *p* ≤ 0.05). These analyzes were performed using Minitab 18.

## Figures and Tables

**Table 1:** Evaluation of the antifungal effect of cell-free extracts of *P. fluorescens* on mycelial growth and the inhibition of A. *alternata* and *F. solani*.

		Phytopathogens			
		*A. alternata*		*F. solani*	
Controls and Incubation Time (h) of *P. fluorescens*	Biomass Dry Weight (g/L)	Mycelial Growth * (cm)	Inhibition (%)	Mycelial Growth * (cm)	Inhibition (%)
Distilled water	NA	5.50 ± 0.00 ^a^	0.00	5.50 ± 0.00 ^a^	0.00
Captan^®^ 0.40 %	NA	4.23 ± 0.10 ^cde^	23.09	4.31 ± 0.17 ^bc^	21.64
2	1.09 ± 0.05	5.20 ± 0.00 ^ab^	5.45	5.17 ± 0.06 ^ab^	6.00
4	1.74 ± 0.12	5.20 ± 0.00 ^ab^	5.45	5.18 ± 0.04 ^ab^	5.82
6	2.47 ± 0.03	5.15 ± 0.00 ^ab^	6.36	5.20 ± 0.00 ^ab^	5.45
8	2.94 ± 0.09	5.13 ± 0.00 ^ab^	6.73	5.20 ± 0.00 ^ab^	5.45
10	3.58 ± 0.03	5.10 ± 0.00 ^bc^	7.27	5.18 ± 0.04 ^ab^	5.82
18	5.08 ± 0.02	4.90 ± 0.00 ^bc^	10.91	4.89 ± 0.26 ^abc^	11.09
24	6.39 ± 0.03	4.37 ± 0.00 ^cd^	20.55	4.80 ± 0.43 ^abc^	12.73
30	6.88 ± 0.07	4.22 ± 0.03 ^de^	23.27	4.55 ± 0.7 ^bcd^	17.27
42	7.02 ± 0.03	3.86 ± 0.36 ^e^	29.82	4.40 ± 0.32 ^cd^	20.00
48	7.31 ± 0.03	3.36 ± 0.11 ^f^	38.91	4.48 ± 0.18 ^cd^	18.55
54	7.58 ± 0.01	3.4 ± 0.06 ^f^	38.18	4.49 ± 0.02 ^cd^	18.36
72	7.75 ± 0.03	3.03 ± 0.24 ^g^	44.91	4.31 ± 0.17 ^d^	21.64
96	8.9 ± 0.02	2.87 ± 0.17 ^gh^	47.82	4.07 ± 0.38 ^d^	26.00
120	8.88 ± 0.01	2.47 ± 0.41 ^gh^	55.09	3.26 ± 0.22 ^e^	40.73
168	8.93 ± 0.08	2.30 ± 0.19 ^h^	58.18	3.09 ± 0.41 ^e^	43.82

* Different letters per column indicate a significant difference (*p* < 0.05) according to Tukey′s comparison of means; N = 6, ± standard deviation.

**Table 2:** Retention factors (Rf) of cell-free extracts of *P. fluorescens*.

Sample	Rf
CE1	0.16
CE2	0.26
CE3	0.61
CE4	0.80
Ph	0.61
DAGP	0.17

Concentrations of standards [0.5 mg mL^−1^] and cell-free extracts of *P. fluorescens* (CE) [1 mg mL^−1^]. Hexane system: acetone (2:1). Read on UV at 245 nm.

**Table 3:** In vitro antifungal activity of the cell-free extracts of *P. fluorescens* and chitosan, alone and combined, on *A. alternata* and *F. solani* development.

	*A. alternata*		*F. solani*	
Treatments	Mycelial Growth * (cm)	Inhibition (%)	Mycelial Growth * (cm)	Inhibition (%)
Distilled water	5.50 ± 0.00 ^a^	0.00	5.50 ± 0.00 ^a^	0.00
Captan^®^ (0.25% *p*/*v*)	4.23 ± 0.10 ^b^	23.09	4.31 ± 0.17 ^b^	21.64
Chitosan 1.5%	3.67 ± 0.17 ^c^	33.27	4.72 ± 0.14 ^b^	14.18
Cell-free extracts of *P. fluorescens* (sample at 120 h)	2.47 ± 0.41 ^d^	55.09	3.26 ± 0.22 ^c^	40.73
Combination of cell-free extracts of *P. fluorescens* with chitosan [50%–1.5% (*v*/*v*)]	2.19 ± 0.06 ^d^	60.18	3.06 ± 0.10 ^c^	44.36

* Different letters per column indicate a significant difference (*p* < 0.05) according to Tukey′s comparison of means; N = 6, ± standard deviation.

## Data Availability

All data needed to evaluate the conclusions in the paper are present in the paper. Additional data related to this paper may be requested from the authors.

## Sample Availability

Samples of the compounds are available from the authors.
